# Juvenile Doxorubicin Exposure Causes Lasting Trabecular Bone Loss in Mice: A Preclinical Model of Long-Term Skeletal Damage

**DOI:** 10.3390/cancers18091438

**Published:** 2026-04-30

**Authors:** Veli Kaan Aydın, Aliye Uysal, Gülçin Abban Mete, Gergana Lengerova, Martina Bozhkova, Steliyan Petrov, Aylin Köseler

**Affiliations:** 1 Department of Biophysics, Faculty of Medicine, Pamukkale University, 20160 Denizli, Türkiye; 2 Department of Histology, Faculty of Medicine, Pamukkale University, 20160 Denizli, Türkiye; aliyeu@pau.edu.tr (A.U.); gabban@pau.edu.tr (G.A.M.); 3 Department of Medical Microbiology and Immunology “Prof. Dr. Elissay Yanev”, Medical University of Plovdiv, 4002 Plovdiv, Bulgaria; gergana.lengerova@mu-plovdiv.bg (G.L.); marti-na.bozhkova@mu-plovdiv.bg (M.B.); steliyan.petrov@mu-plovdiv.bg (S.P.); 4 Research Institute, Medical University of Plovdiv, 4002 Plovdiv, Bulgaria

**Keywords:** doxorubicin, osteotoxicity, microarchitecture, remodeling, biomarkers, cartilage, trabeculae, survivorship

## Abstract

Background/Objectives: Anthracyclines such as doxorubicin (DOX) are integral to pediatric cancer protocols, yet little is known about how juvenile DOX exposure shapes the long-term trajectory of bone growth, microarchitectural connectivity, and the functional balance of bone turnover after treatment cessation. This study aimed to define how juvenile DOX exposure remodels trabecular architecture and bone homeostasis both acutely and after recovery. Methods: Four-week-old female BALB/c mice were treated with 6 mg/kg DOX or saline once weekly for four weeks. Bone parameters were analyzed immediately after treatment and after a 4-week drug-free recovery period. Assessments included high-resolution µCT for bone structure and connectivity, H&E and TRAP staining for histological evaluation, and ELISA for bone turnover markers (PINP, OC/BGP, TRACP-5b) in both serum and bone marrow. Results: DOX exposure significantly compromised trabecular bone mass and network connectivity, with persistent bone loss extending into the recovery period. Histologically, DOX caused marked degeneration in the epiphyseal growth plate and calcified zones, alongside a marked increase in osteoclast numbers. Functionally, an acute increase in circulating bone formation markers was observed post-treatment. However, during the recovery phase, this transitioned to a significant suppression of these systemic markers, coupled with significantly increased localized bone resorption. Conclusions: Juvenile DOX exposure produces sustained trabecular network impairment and growth plate degeneration. This durable structural deterioration is functionally associated with the establishment of a localized, pathologically uncoupled remodeling environment.

## Simple Summary

Childhood cancer treatments save lives, but some drugs, like doxorubicin, can cause long-lasting damage to growing bones. This study investigates how short-term doxorubicin exposure during a critical growth period affects bone structure and cellular health over time in a mouse model. We aimed to understand if the skeleton can recover after treatment ceases and how the bone remodeling process responds. We found that the drug causes substantial, sustained loss of spongy bone, damages the cartilage necessary for bone lengthening, and creates a lasting pathological imbalance where bone resorption significantly outpaces circulating markers of bone formation. These findings help explain why pediatric cancer survivors frequently suffer from persistent bone fragility and fractures, highlighting the critical need to develop targeted protective strategies for their skeletal health during and after cancer therapy.

## 1. Introduction

Chemotherapy remains a cornerstone of cancer care, improving survival across malignancies, yet its physiological toxicities significantly compromise patients’ long-term quality of life [1,2]. Skeletal toxicity—manifesting as loss of bone mineral density (BMD), disrupted trabecular microarchitecture, and increased fracture risk—has gained increasing recognition over the past decades [1,2]. Although survival gains have been emphasized, the musculoskeletal consequences, particularly in growing children, are often underappreciated [3,4]. Early alerts from pediatric survivor cohorts linked anthracycline exposure to reduced bone density, growth impairment, and fractures [5], and subsequent studies in childhood acute lymphoblastic leukemia (ALL) survivors confirmed lower lumbar spine and hip BMD, and higher rates of osteopenia/osteoporosis compared with peers. These deficits represent sustained complications that may extend well into adulthood [6,7,8].

Anthracyclines such as doxorubicin (DOX) are integral to protocols for childhood ALL/AML, Wilms tumor, Ewing sarcoma, osteosarcoma, and soft-tissue sarcomas, appearing in a large fraction of pediatric regimens [9]. DOX intercalates DNA and inhibits topoisomerase II, causing double-strand breaks; its quinone moiety undergoes redox cycling, generating increased amounts of reactive oxygen species (ROS) that damage macromolecules [10,11]. While cardiotoxicity is the best-known dose-limiting toxicity [12], converging evidence indicates direct and increased skeletal injury. In preclinical models, DOX decreases trabecular bone volume and worsens osteolysis via oxidative stress and TGF-β signaling, suppresses osteoblast survival and differentiation, and enhances osteoclast activity—mechanisms that can be partially rescued by antioxidants or TGF-β blockade [13,14]. Additional studies show compromised bone integrity in rats [15] and sustained, delayed bone loss after a single juvenile DOX exposure in mice [16]. At the cellular level, DOX can impair osteoblastogenesis from mesenchymal stromal cells and favor adipogenic drift [17,18,19], while recent work implicates a mitochondrial ROS–TRPML1–TFEB autophagy axis that augments osteoclastic bone resorption [20]. Together, these data suggest that DOX perturbs both arms of bone remodeling.

Despite these signals, most preclinical work emphasizes the immediate post-treatment window, and clinical survivor studies seldom isolate DOX effects from multiagent therapy [21]. Critically, little is known about how juvenile DOX exposure—occurring during the peak period of skeletal accrual [22]—shapes the long-term trajectory of bone growth, microarchitectural integrity, and marrow stress pathways after treatment cessation. Addressing this gap has direct pediatric relevance, as adolescent and young adult survivors exposed to anthracyclines exhibit impaired skeletal accrual and persistent fragility well beyond the completion of their therapy [7,8].

To address this, we utilized a juvenile mouse model to quantify both the acute and sustained consequences of DOX on the skeletal system. We paired high-resolution µCT—incorporating advanced connectivity-sensitive metrics—with detailed histological evaluations of the growth plate and trabecular network. Furthermore, we assessed circulating and localized bone turnover markers (PINP, OC/BGP, and TRACP-5b in serum and bone marrow) to evaluate the functional status of bone remodeling. By defining how juvenile DOX exposure disrupts trabecular architecture and contributes to an imbalance in bone turnover, this study aims to inform supportive clinical strategies and the timing of interventions to mitigate long-term skeletal damage in pediatric cancer survivors.

## 2. Materials and Methods

### 2.1. In Vivo Studies

Female, four-week-old BALB/c mice were obtained from the Pamukkale University Experimental Animal Research Center (Denizli, Turkey). The rationale for selecting female mice stems from their established utilization in preclinical doxorubicin osteotoxicity models and specific sex-dependent variations in bone adaptation during the developmental period [4]. The animals were housed under standard laboratory conditions (22 ± 2 °C, 12 h light/dark cycle) with unrestricted access to standard chow and water. All experimental procedures were approved by the Pamukkale University Animal Experiments Local Ethics Committee (Protocol Number: PAUHDEK-2024/16) and conducted in strict accordance with national animal welfare guidelines at a facility licensed by the Turkish Ministry of Agriculture and Forestry.

**Figure 1:**
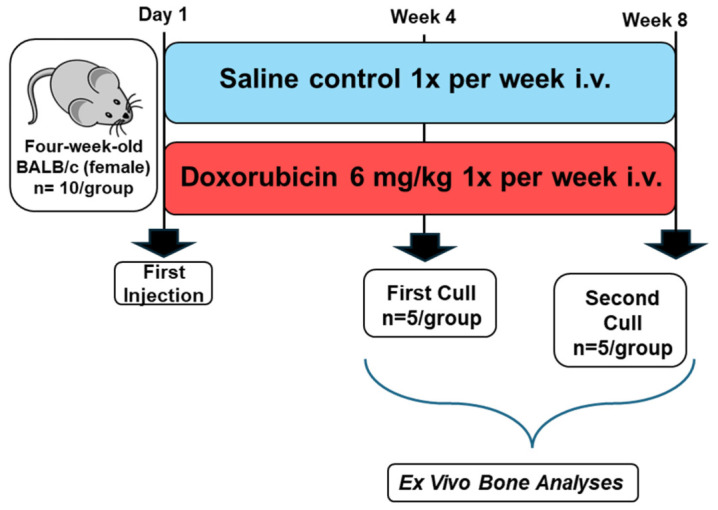
Experimental Summary. Four-week-old female BALB/c mice (*n* = 10/group) were treated with either doxorubicin (6 mg/kg) or saline once a week via tail vein injection. After 4 weeks’ treatment schedule half of the animals per group were sacrificed while rest housed for recovery. At the end of the 8th week remaining animals were culled, bone and tissue samples were collected, and ex vivo analyses were carried out.

To evaluate both the immediate and prolonged skeletal impacts of DOX, the mice (*n* = 10/group) were intravenously administered either 100 µL of sterile saline (control) or 6 mg/kg DOX (clinical grade, Koçak Farma, İstanbul, Turkey) once weekly via the tail vein (Figure 1). Following the 4-week treatment regimen, half of the animals from each group (*n* = 5/group) were euthanized for immediate ex vivo analyses. The remaining cohort was maintained for an additional 4-week drug-free recovery period and sacrificed at the end of week 8. Peripheral blood was collected for serum extraction, and the hind limb bones (tibiae and femora) were immediately harvested. Bones destined for microcomputed tomography (µCT) and histological evaluations were fixed in 4% paraformaldehyde, whereas fresh tibiae were utilized for marrow extraction.

### 2.2. Microcomputed Tomography (µCT) Analysis

Microarchitectural parameters of the isolated tibiae were evaluated utilizing a desktop SkyScan 1275 µCT system (Bruker, Aartselaar, Belgium). High-resolution scanning was performed applying a 0.5 mm aluminum filter with an isotropic voxel size of 4.3 µm. The rotational step was set to 0.7° covering a full 180° scan trajectory. Two-dimensional projections were reconstructed into 3D datasets using NRecon software (v 1.7.4.2).

Quantitative morphometry of the trabecular and cortical regions was conducted via CTan software (v 1.18.8.0). The region of interest (ROI) for trabecular bone was delineated starting 0.2 mm below the growth plate (spongy bridge) and extending 1.0 mm longitudinally into metaphysis. For cortical bone, the offset was set to 1.0 mm from the reference point. Following ROI selection, a standardized batch analysis protocol—comprising “Thresholding” (optimized between 80–255), “Despeckle” (removal of noise < 10 voxels), and “3D Analysis”—was uniformly applied across all samples to compute trabecular bone volume, number, thickness, separation, and connectivity indices.

### 2.3. Histological Processing and TRAP Staining

Extracted tibiae were fixed in 4% paraformaldehyde (Sigma-Aldrich, St. Louis, MO, USA) for 48 h and subsequently decalcified using a 0.5 M EDTA solution (pH 7.4; Sigma-Aldrich, St. Louis, MO, USA) supplemented with 0.5% paraformaldehyde for 14 days. Completely decalcified specimens underwent standard automated tissue processing and were embedded in paraffin wax. To assess the epiphyseal growth plate and the trabecular microenvironment, 3 µm thick longitudinal sections were obtained using a microtome. These sections were primarily stained with Hematoxylin and Eosin (H&E; Sigma-Aldrich, St. Louis, MO, USA) following standard laboratory protocols [23] to evaluate general cellular organization and structural integrity. Furthermore, Tartrate-Resistant Acid Phosphatase (TRAP) staining was performed on consecutive longitudinal sections using an azo dye coupling method to specifically identify and quantify multinucleated osteoclasts. Briefly, deparaffinized sections were incubated in an acetate-tartrate buffer (pH 5.2) containing sodium tartrate dihydrate (Sigma-Aldrich, St. Louis, MO, USA). The enzymatic reaction was visualized using a substrate solution of naphthol AS-BI phosphate (Sigma-Aldrich) dissolved in dimethylformamide (Fisher Scientific, Waltham, MA, USA), followed by coupling with hexazotized pararosaniline—prepared from pararosaniline and sodium nitrite (Sigma-Aldrich)—to form a distinct red precipitate within the osteoclast cytoplasm. Finally, the sections were counterstained with Gill’s hematoxylin, dehydrated through graded ethanols, cleared in xylene, and mounted with DPX.

### 2.4. Bone Marrow Fluid Extraction

Freshly isolated tibiae were immediately chilled in ice-cold phosphate-buffered saline (PBS; Sigma-Aldrich, St. Louis, MO, USA) and sanitized through sequential washes in 100% and 70% ethanol. To expose the medullary cavity, only the proximal epiphyses of the tibiae were carefully excised using sterile scissors, leaving the distal ends intact. The resulting diaphyseal bone shafts were placed vertically—with the cut proximal end facing downward—inside pierced 0.2 mL microcentrifuge tubes. These were nested within larger 1.5 mL collection tubes containing 200 µL of sterile PBS supplemented with 100 U/mL penicillin and 100 µg/mL streptomycin (Sigma-Aldrich, St. Louis, MO, USA). Rather than utilizing a traditional fluid flushing technique, the nested assembly was centrifuged at 6000 rpm for 5 min. The mechanical centrifugal force effectively expelled the entire bone marrow pellet out of the single open end of the diaphyseal cavity directly into the PBS reservoir below. The extruded marrow was then gently pipetted to create a homogeneous suspension for subsequent biochemical measurements.

### 2.5. Enzyme-Linked Immunosorbent Assay (ELISA)

Serum and bone marrow fluid concentrations of specific bone turnover markers were quantified utilizing commercially available ELISA kits (Elabscience, Wuhan, China) according to the manufacturer’s operational guidelines. The targeted markers included procollagen I N-terminal propeptide (PINP, E-EL-M0233), osteocalcin (OC/BGP, E-EL-M0864) for bone formation, and tartrate-resistant acid phosphatase 5b (TRACP-5b, E-EL-M3100) for bone resorption.

### 2.6. Statistical Analysis

Data visualization and statistical analyses were performed using GraphPad Prism software (Version 10.4; GraphPad Software, Boston, MA, USA). The distribution of variables was assessed, and group comparisons were subsequently conducted utilizing the non-parametric Mann–Whitney U test due to the sample size. Statistical significance thresholds were established at *p* < 0.05. Precise *p*-values are reported in the text, and significance levels in figures are denoted as * *p* < 0.05, ** *p* < 0.01, *** *p* < 0.001, and **** *p* < 0.0001.

## 3. Results

### 3.1. Juvenile DOX Exposure Causes Significant Trabecular Bone Loss

**Figure 2:**
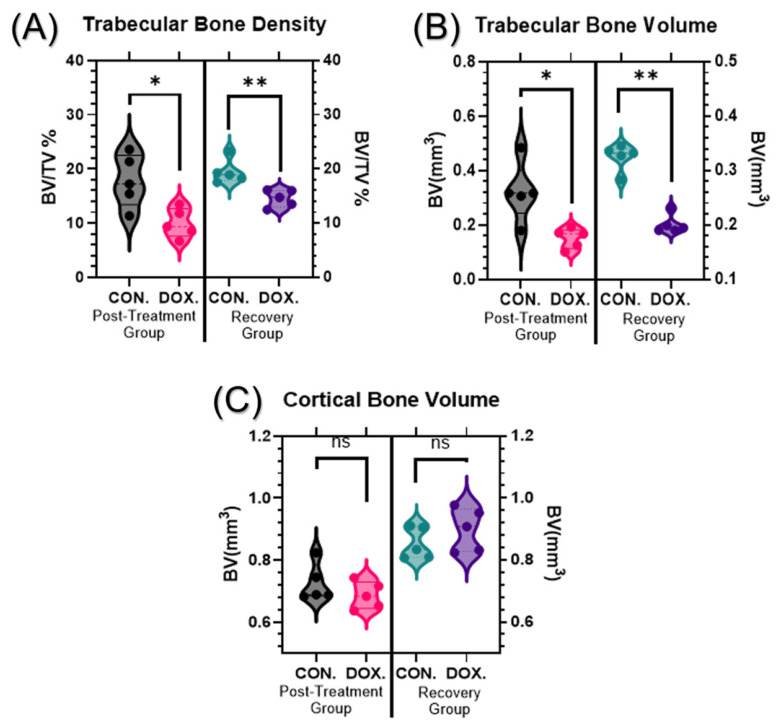
Effects of juvenile DOX exposure on bone mass and volume parameters. (**A**) Trabecular bone density (bone volume/tissue volume, BV/TV %), (**B**) trabecular bone volume (BV, mm^3^), and (**C**) cortical bone volume (BV, mm^3^) of the tibiae. Female BALB/c mice (four-week-old) were treated with either saline (control) or DOX (6 mg/kg) once weekly for 4 weeks. Animals were sacrificed at 4 weeks (acute effects) or maintained for an additional 4 weeks without treatment (8 weeks total, long-term effects). Bone parameters were analyzed using micro-computed tomography (μCT). Data are presented as violin plots showing distribution of values. Statistical significance was determined using Mann–Whitney test (* *p* ≤ 0.05, ** *p* ≤ 0.01, ns: not significant).

Micro-computed tomography (µCT) analysis revealed that juvenile DOX exposure significantly compromised trabecular bone mass and volume parameters. Trabecular bone density (BV/TV) was markedly reduced in DOX-treated animals compared to controls in both the post-treatment and recovery groups. In the post-treatment group, DOX-treated animals exhibited a 43.98% decrease in trabecular bone density (17.78 ± 4.84% vs. 9.96 ± 2.69%, *p* = 0.0317). This increased reduction was sustained in the recovery group, demonstrating a 25.05% decrease (19.44 ± 2.23% vs. 14.57 ± 1.60%, *p* = 0.0079) (Figure 2A).

**Figure 3:**
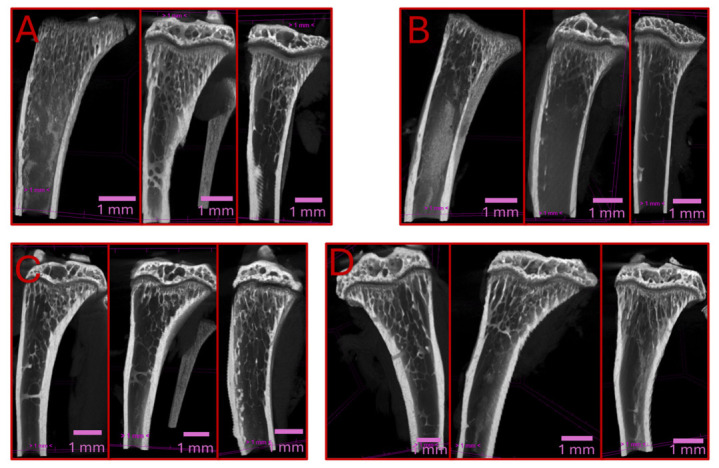
Representative µCT reconstructions of the proximal tibia. Representative images from three distinct, randomly selected biological samples per group are presented for: (**A**) Control group immediately post-treatment; (**B**) DOX-treated group immediately post-treatment; (**C**) Control group after the 4-week recovery phase; and (**D**) DOX-treated group after the 4-week recovery phase. To ensure objective morphological comparison, all 3D visualizations were generated using CTVox software (v 3.3.0 r1403; Bruker, Aartselaar, Belgium) applying identical thresholding levels and standardized anatomical orientations across all cohorts. Note the increased trabecular depletion in both DOX-treated groups (**B**,**D**) compared to their respective age-matched controls (**A**,**C**). Scale bars = 1 mm.

Trabecular bone volumes were similarly reduced following DOX treatment. Post-treatment analysis revealed a 52.8% decrease in DOX-treated animals (0.320 ± 0.110 mm^3^ vs. 0.151 ± 0.037 mm^3^, *p* = 0.0160), with persistent bone loss observed in the recovery group (37.5% reduction, 0.320 ± 0.024 mm^3^ vs. 0.200 ± 0.017 mm^3^, *p* = 0.0080) (Figure 2B). Conversely, cortical bone volume remained largely unaffected by the DOX regimen, showing no significant differences between the groups in either the post-treatment (0.72 ± 0.06 mm^3^ vs. 0.69 ± 0.04 mm^3^, *p* = 0.3100) or recovery periods (0.85 ± 0.05 mm^3^ vs. 0.90 ± 0.07 mm^3^, *p* = 0.3100) (Figure 2C). Representative 3D µCT reconstructions (Figure 3) visually corroborate these findings, clearly illustrating the increased trabecular depletion in DOX-treated samples, particularly immediately post-treatment.

### 3.2. DOX Treatment Alters Trabecular Bone Architecture

**Figure 4:**
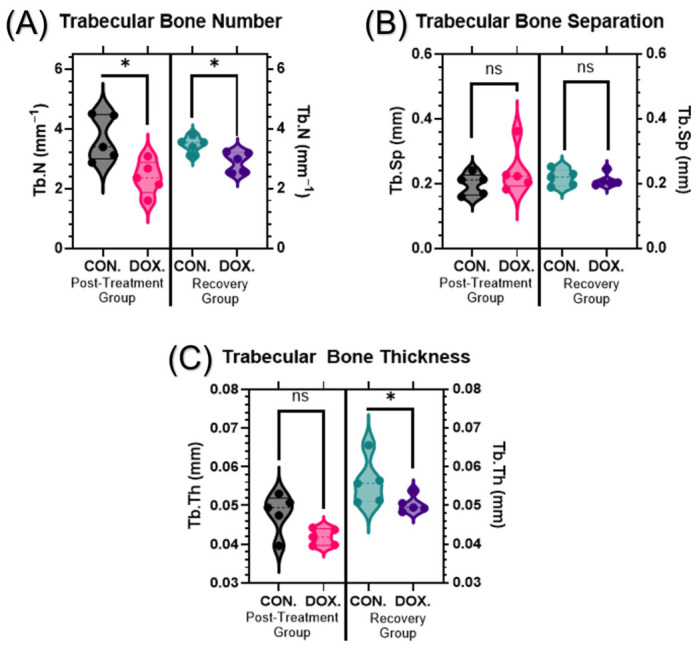
DOX-induced changes in trabecular bone architecture. (**A**) Trabecular bone number (Tb.N, mm^−1^), (**B**) trabecular bone separation (Tb.Sp, mm), and (**C**) trabecular bone thickness (Tb.Th, mm) Trabecular bone architectural parameters were analyzed following juvenile DOX exposure. Mice were treated as described in Figure 1. Trabecular bone number, thickness, and separation were quantified from μCT scans. Violin plots show the distribution of architectural parameters across treatment groups and timepoints. Statistical analysis was performed using Mann–Whitney test (* *p* ≤ 0.05, ns: not significant).

Analysis of trabecular bone architectural parameters revealed significant structural alterations following juvenile DOX exposure. Trabecular bone number (Tb.N) was significantly reduced in DOX-treated animals, showing a 28.4% decrease in the post-treatment group (3.67 ± 0.76 mm^−1^ vs. 2.37 ± 0.56 mm^−1^, *p* = 0.0160) and a 15.2% reduction in the recovery group (3.48 ± 0.25 mm^−1^ vs. 2.90 ± 0.34 mm^−1^, *p* = 0.0320) (Figure 4A).

No significant changes were found in trabecular separation (Tb.Sp) associated with DOX treatment in either the post-treatment (0.198 ± 0.033 mm vs. 0.240 ± 0.070 mm, *p* = 0.4200) or recovery groups (0.217 ± 0.025 mm vs. 0.210 ± 0.026 mm, *p* = 0.8400) (Figure 4B). While trabecular thickness (Tb.Th) did not show a statistically significant difference between groups immediately post-treatment (0.048 ± 0.005 mm vs. 0.041 ± 0.002 mm, *p* = 0.0950), the recovery group exhibited a significant 10.7% decrease in thickness (0.056 ± 0.006 mm vs. 0.050 ± 0.002 mm, *p* = 0.0317) (Figure 4C).

### 3.3. DOX Exposure Compromises Structural Connectivity and Bone Quality

**Figure 5:**
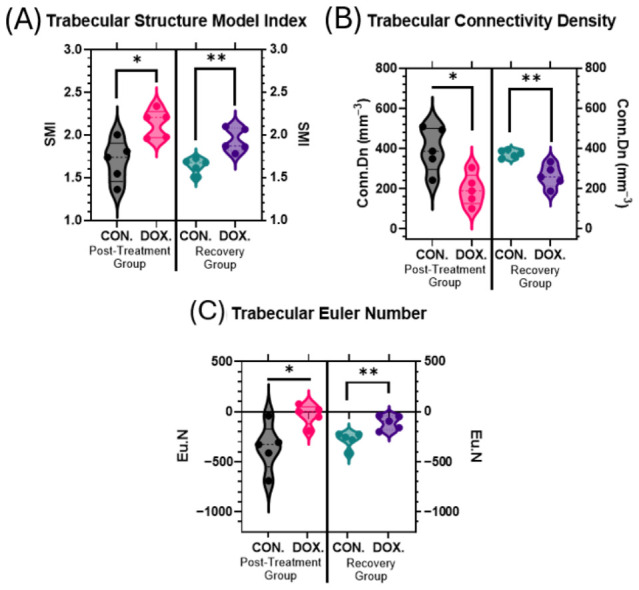
Structural connectivity and bone quality alterations following DOX treatment. (**A**) Trabecular Structure Model Index (SMI), (**B**) trabecular connectivity density (Conn.Dn, mm^−3^), and (**C**) trabecular Euler number (Eu.N). Structural connectivity and bone quality parameters were assessed to evaluate the long-term impact of juvenile DOX exposure. Structure Model Index (SMI), connective density, and Euler number were calculated from μCT data. Violin plots demonstrate the distribution of structural parameters. Statistical significance was determined using Mann–Whitney test (* *p* ≤ 0.05, ** *p* ≤ 0.01).

Structural connectivity and bone quality parameters demonstrated significant degradation following juvenile DOX treatment. The Structure Model Index (SMI) significantly increased in DOX-treated animals, indicating a structural shift from a plate-like to a structurally weaker rod-like trabecular architecture. Post-treatment analysis revealed a 26.5% increase in SMI (1.69 ± 0.25 vs. 2.13 ± 0.16, *p* = 0.0317), a trend that persisted with an 18.3% increase during the recovery phase (1.64 ± 0.09 vs. 1.94 ± 0.14, *p* = 0.0079) (Figure 5A).

Connectivity density (Conn.Dn) was markedly reduced in DOX-treated animals, directly reflecting diminished trabecular network integrity. Post-treatment analysis showed a 50.7% decrease (396.8 ± 109.6 mm^−3^ vs. 195.4 ± 77.47 mm^−3^, *p* = 0.0160), with a sustained 29.8% reduction observed in the recovery group (374.8 ± 18.83 mm^−3^ vs. 263.2 ± 55.48 mm^−3^, *p* = 0.0079) (Figure 5B). Furthermore, the Euler number (Eu.N), an indicator of topological connectivity, was significantly elevated (less negative) in DOX-treated animals for both the post-treatment (−354.4 ± 233.4 vs. −29.0 ± 101.6, *p* = 0.0310) and recovery groups (−280.0 ± 79.26 vs. −108.4 ± 70.35, *p* = 0.0079), confirming substantial trabecular disconnection and structural fragmentation (Figure 5C).

### 3.4. DOX Treatment Induces Histological Degeneration in the Growth Plate and Trabecular Network

**Figure 6:**
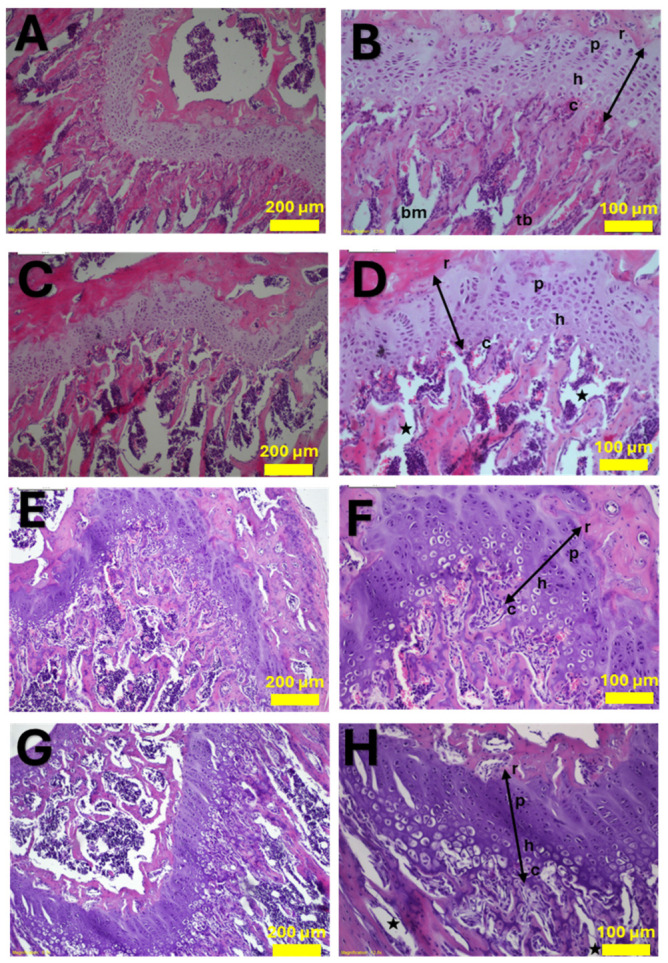
Histological assessment of doxorubicin-induced growth plate and trabecular degeneration. Representative H&E-stained sections of the proximal tibia. (**A**,**B**) Control group post-treatment; (**C**,**D**) DOX-treated group post-treatment; (**E**,**F**) Control group after recovery phase; (**G**,**H**) DOX-treated group after recovery phase. The left column (**A**,**C**,**E**,**G**) displays low-magnification overviews of the metaphysis and trabecular network. The right column (**B**,**D**,**F**,**H**) provides high-magnification details of the chondro-osseous junction. Healthy cartilage zones are indicated by double arrows in control groups: reserve zone (r), proliferation zone (*p*), hypertrophic zone (h), and calcified zone (c). Trabecular bone (tb) and bone marrow (bm) are marked. Stars (*) specifically indicate the pathological degenerative spaces (gaps) formed between the cartilage and bone trabeculae in DOX-treated groups. Scale bars represent 200 µm in the left column and 100 µm in the right column.

Histological evaluation (H&E staining) of the proximal tibia revealed profound structural alterations following DOX treatment (Figure 6). In the post-treatment control group, the growth plate and trabecular bone exhibited normal anatomical organization. The distinct cartilage zones (reserve, proliferative, hypertrophic, and calcified) were well-defined, and the underlying trabecular network was structurally sound and continuously connected to the chondro-osseous junction.

Conversely, the post-treatment DOX group displayed a severe loss of normal cellular arrangement within the growth plate tissue. Cells in the reserve zone appeared swollen and hypertrophic. Notably, marked degeneration in the calcified cartilage zone led to prominent structural gaps, disrupting the critical connection between the cartilage and the underlying primary spongiosa. Furthermore, the majority of the trabecular bone connections were physically broken, resulting in irregularly expanded bone marrow spaces.

Following the recovery phase, the control group maintained generally normal morphology, despite minor age-related structural adaptations. However, the DOX-treated recovery group exhibited even more severe, sustained cartilage degeneration. The reserve, proliferation, and hypertrophy zones lost their organized columnar structures and appeared notably thinned. The prominent gaps between the calcified cartilage and bone tissue persisted, indicating a failure of normal endochondral ossification. In this group, the trabecular bone network was found reduced, presenting only as small, isolated spicules within the pathologically expanded marrow cavity.

### 3.5. DOX Exposure Significantly Increases Localized Osteoclast Numbers

**Figure 7:**
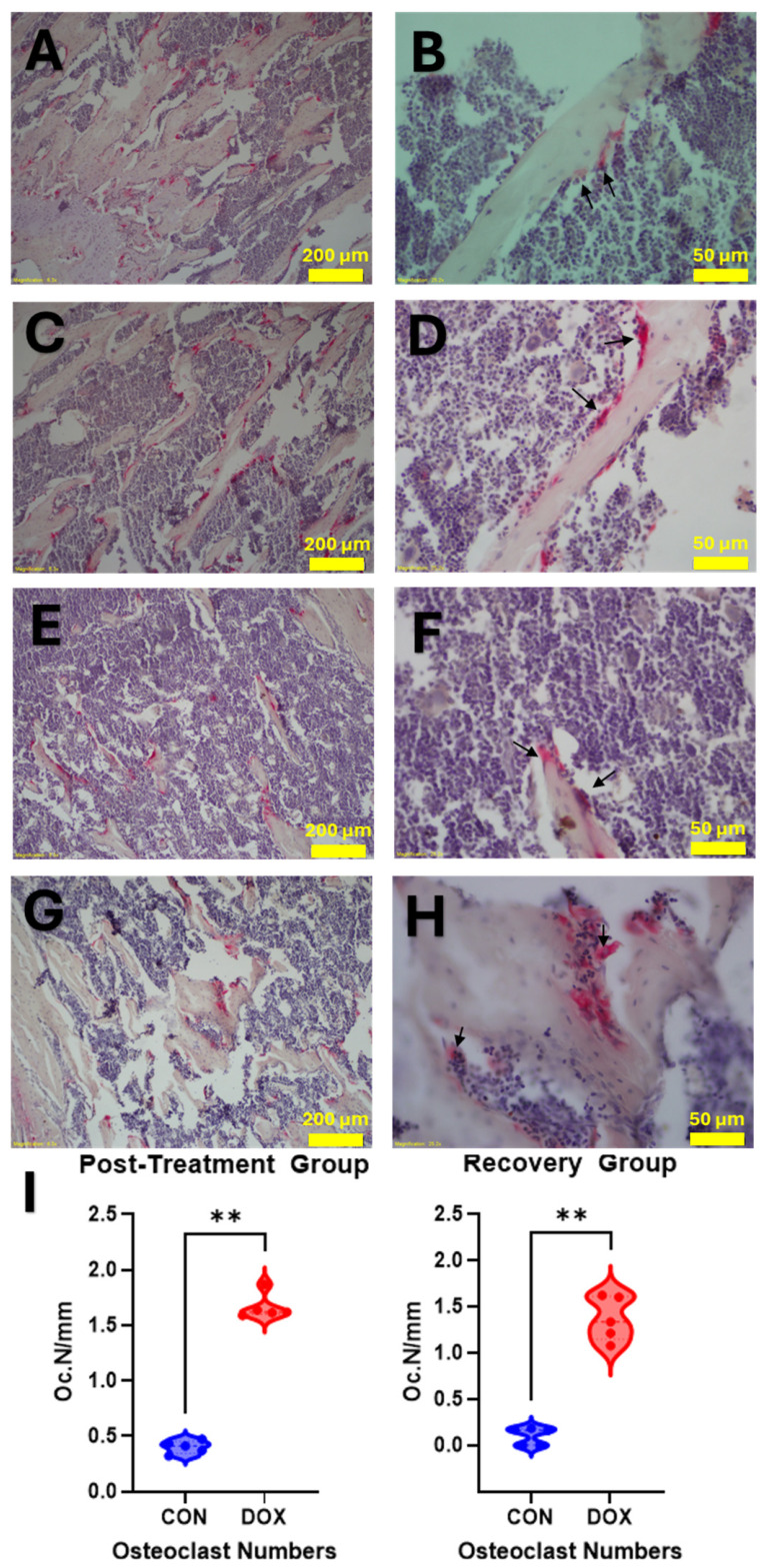
Visualization and quantification of doxorubicin-induced osteoclast upregulation. Tartrate-resistant acid phosphatase (TRAP) stained sections of the proximal tibia. (**A**,**B**) Control group post-treatment; (**C**,**D**) DOX-treated group post-treatment; (**E**,**F**) control group after recovery phase; (**G**,**H**) DOX-treated group after recovery phase. The left column displays lower-magnification overviews, while the right column shows higher-magnification details of trabecular bone and marrow spaces. Black arrows indicate highly active, multinucleated TRAP-positive osteoclasts (stained red) predominantly residing on bone surfaces in DOX-treated groups. (**I**) Quantitative histomorphometric analysis of osteoclast number per bone perimeter (Oc.N/mm). Blue and red violins represent the control and DOX-treated mice, respectively. Median values are presented. DOX exposure significantly increases localized osteoclast density both acutely and after the recovery period. ** *p* < 0.01 (Mann–Whitney U test). Scale bars represent 200 µm in the left column and 50 µm in the right column.

To functionally validate the localized trabecular bone loss and morphological degradation observed, TRAP staining was performed to quantify active, multinucleated osteoclasts residing on the bone surfaces (Figure 7).

Quantitative analysis revealed a significant increase in osteoclast numbers (Oc.N/mm) in DOX-treated animals. Immediately post-treatment, the median osteoclast number was significantly elevated in the DOX group compared to controls (1.619 Oc.N/mm vs. 0.412 Oc.N/mm, *p* = 0.0079). Critically, this heightened resorptive cellular presence was not merely a transient acute response; the significant increase in osteoclasts was robustly maintained during the recovery phase (1.337 Oc.N/mm vs. 0.161 Oc.N/mm, *p* = 0.0079). These histological findings provide direct cellular evidence of a sustained, DOX-driven hyper-resorptive environment within the trabecular compartment.

### 3.6. Doxorubicin Alters Bone Turnover Markers in Serum and Bone Marrow

**Figure 8:**
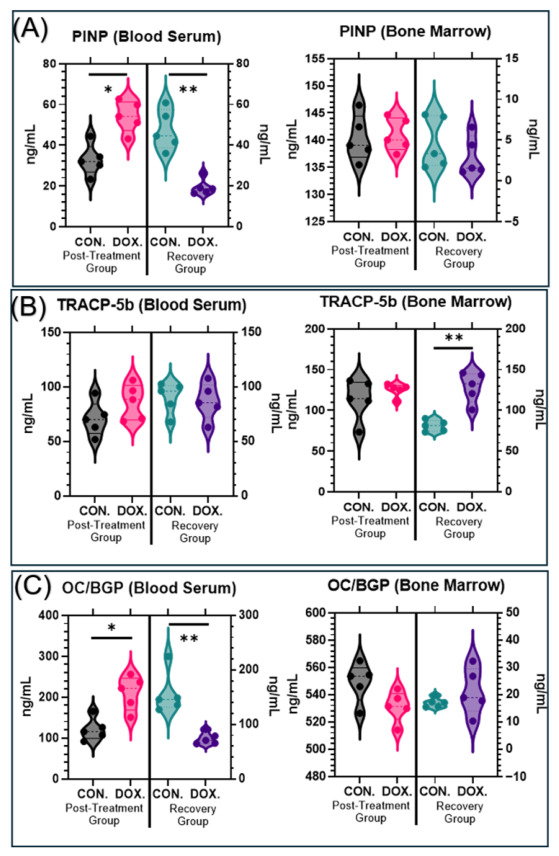
Doxorubicin (DOX) treatment alters bone turnover markers in blood serum and bone marrow. Violin plots comparing the concentrations (ng/mL) of key bone turnover markers in control (CON.) vs. doxorubicin-treated (DOX.) groups. Samples were analyzed at two time points: a “Post-Treatment” group and a “Recovery” group. For each marker, measurements were taken from both blood serum (left-side plots) and bone marrow (right-side plots). (**A**) Procollagen type I N-terminal propeptide (PINP), a marker for bone formation. (**B**) Osteocalcin/Bone Gla protein (OC/BGP), a marker associated with bone formation. (**C**) Tartrate-resistant acid phosphatase 5b (TRACP-5b), a marker for bone resorption. Data are presented as violin plots, which show the median (white dot), interquartile range (thick inner line), and the full distribution of data points. Black and pink violins represent the control and DOX-treated mice in the post-treatment group, respectively. Teal and purple violins represent the control and DOX-treated mice in the recovery group, respectively. Asterisks denote a statistically significant difference between the CON. and DOX. groups within that time point (* *p* < 0.05, ** *p* < 0.01).

To further delineate the impact of DOX on systemic and localized bone homeostasis, key bone turnover markers were quantified via ELISA (Figure 8). The bone formation markers procollagen type I N-terminal propeptide (PINP) and osteocalcin (OC/BGP) demonstrated a distinct biphasic pattern in the blood serum. In the post-treatment group, serum levels of both PINP (32.84 ± 7.66 ng/mL vs. 54.19 ± 7.65 ng/mL, *p* = 0.0160) and OC/BGP (121.2 ± 27.69 ng/mL vs. 210.5 ± 41.88 ng/mL, *p* = 0.0160) were significantly elevated in the DOX-treated cohort compared to controls. This suggests an acute, albeit structurally ineffective, surge in bone formation activity immediately following treatment.

However, this trend was found reversed during the recovery period. Serum concentrations of both PINP (47.44 ± 9.97 ng/mL vs. 19.46 ± 3.94 ng/mL, *p* = 0.0080) and OC/BGP (156.5 ± 39.19 ng/mL vs. 74.26 ± 11.02 ng/mL, *p* = 0.0080) were significantly suppressed in the DOX group, consistent with a delayed reduction in circulating bone formation markers. In contrast to the serum, no significant differences were detected in the concentrations of PINP (*p* = 0.6900 post-treatment; *p* = 0.2200 recovery) or OC/BGP (*p* = 0.0952 post-treatment; *p* = 0.3095 recovery) in the localized bone marrow fluid at either time point.

When assessing bone resorption via tartrate-resistant acid phosphatase 5b (TRACP-5b), no significant differences were observed in the blood serum between the groups at either time point (*p* = 0.2200 post-treatment; *p* = 0.6900 recovery). However, aligning with our histological osteoclast counts, a distinct effect was noted within the local bone marrow microenvironment. While marrow TRACP-5b levels did not significantly differ immediately post-treatment (113.6 ± 25.04 ng/mL vs. 125.0 ± 8.21 ng/mL, *p* > 0.9999), the DOX-treated animals exhibited a significant elevation in localized TRACP-5b concentration during the recovery phase (80.95 ± 6.86 ng/mL vs. 128.7 ± 18.60 ng/mL, *p* = 0.0079).

In summary, the combined structural, histological, and biochemical data indicate that doxorubicin exposure induces a complex, time-dependent disruption of bone turnover. An acute surge in systemic formation markers—without corresponding structural preservation—rapidly transitions into a sustained state characterized by systemic suppression of formation markers alongside a localized, cellularly driven increase in bone resorption.

## 4. Discussion

This study demonstrates that juvenile exposure to doxorubicin (DOX) produces a sustained, trabecular-predominant bone reduction characterized by loss of mass (BV/TV), reduced trabecular number, plate-to-rod conversion (higher SMI), and diminished network integrity (lower connectivity density, higher Euler number), while cortical volume is largely preserved (Figure 2, Figure 4 and Figure 5). These structural signatures were found to persist robustly after a drug-free recovery period, indicating that early-life anthracycline exposure inflicts an architectural injury that extends far beyond acute toxicity. The delayed persistence of microarchitectural deterioration in our model mirrors the clinical reports of long-term skeletal fragility in childhood cancer survivors exposed to anthracyclines [5,6,7,8,24].

Our histological findings provide critical tissue-level corroboration of this architectural injury (Figure 6). The severe degeneration in the growth plate zones—marked by the loss of normal cellular arrangement and profound structural gaps—highlights DOX’s detrimental impact on endochondral ossification. Previous literature, such as the work by van Leeuwen et al. (2003), reported growth plate thinning following chemotherapy [25]. In our juvenile model, we observed severe architectural disorganization acutely, which visibly progressed to noticeable thinning of the proliferative and hypertrophic zones during the recovery phase, effectively reconciling these observations. Given that altered growth plate dynamics fundamentally impair longitudinal bone growth [26], our findings align with the reduced skeletal growth trajectories often observed clinically in pediatric patients. Interestingly, while a recent study utilizing adult mice reported no measurable effects of DOX on macroscopic bone growth [4], the severe disruption observed in our juvenile model emphasizes the high vulnerability of the developing skeleton to chemotherapy-induced osteotoxicity.

A novel insight into the underlying functional response is provided by our bone turnover marker (BTM) analysis. Immediately post-treatment, an unexpected, significant increase in bone formation markers (serum PINP and OC/BGP) was detected. This finding appears paradoxical given the substantial trabecular bone loss observed via µCT at the identical timepoint. This acute mismatch between elevated circulating markers and trabecular loss suggests that the early systemic osteoblastic response—potentially a compensatory effort—is not effectively reflected in local bone mass maintenance. While serum PINP and OC/BGP reflect systemic protein secretion, their elevation at this timepoint does not translate into stable trabecular incorporation, likely due to the overwhelming resorptive activity or a localized failure in functional bone matrix deposition [27]. Further dynamic histomorphometry would be required to definitively map the temporal relationship between this protein surge and effective mineral apposition.

This acute, dysfunctional state transitions into a lasting, pathological uncoupling during the recovery period. Circulating formation signals become significantly suppressed (low serum PINP and OC/BGP), while local bone resorptive activity is heavily upregulated. Crucially, our TRAP staining visually and quantitatively confirmed a significant and sustained upregulation of osteoclast numbers (Oc.N/mm) residing on the trabecular surfaces (Figure 7). This localized, cellularly driven hyper-resorption is highly consistent with recent evidence demonstrating that DOX profoundly modifies multiple cell populations within the bone marrow niche [14] and activates inflammatory pathways that drive bone loss [13]. Furthermore, the eventual suppression of these circulating formation markers during the recovery phase is consistent with a shift toward a low-turnover state. While our data do not directly measure the cellular status of the bone marrow, this systemic decline in bone formation signals aligns with existing literature suggesting that chemotherapy can induce a state of cellular senescence or long-term impairment of the osteoblastic lineage [18,19]. The fact that this pathology presents predominantly in the trabecular compartment is likely due to its inherently higher metabolic activity and remodeling rate [28,29], rendering its distinct cellular populations highly susceptible to DOX cytotoxicity [30,31].

While this experimental design successfully separated acute from sustained defects—an underexplored period critical for peak bone mass recovery [22]—several limitations must be explicitly acknowledged. First, observing a 4-week recovery period provides a critical window into post-treatment dynamics; however, it does not fully replicate the multi-decade lifespan of a childhood cancer survivor. Therefore, our “sustained” effects should be cautiously extrapolated to true chronic clinical timelines. Second, we utilized healthy BALB/c mice rather than a tumor-bearing ALL/AML model. While this design isolates the direct osteotoxic effects of DOX from tumor-induced osteolysis, healthy models may not capture the full complexity of the oncologic microenvironment [21]. Finally, although we successfully quantified osteoclast numbers via TRAP staining, the absence of dynamic histomorphometry and direct osteoblast cell counting limits our ability to fully map the temporal cellular kinetics of bone formation.

These data suggest important translational implications. Clinically, anthracyclines are embedded in many pediatric regimens, and survivor cohorts consistently show persistent BMD deficits and fractures [2,3,5,6]. Our preclinical findings further underscore the importance of early bone health surveillance and support the ongoing implementation of international guidelines, such as DXA monitoring during and after therapy, and optimization of calcium/vitamin D. Mechanism-directed strategies merit pediatric evaluation: antioxidant support, TGF-β pathway modulation, and strictly timed antiresorptive therapies. Preclinical testing should also assess structured mechanical loading and cardioprotectants (e.g., dexrazoxane) for skeletal benefit or neutrality [32]. Finally, it must be acknowledged that while µCT was performed at a representative site, our analysis focused on local trabecular dynamics and circulating markers. Therefore, these findings should be interpreted as indicators of localized microarchitectural injury and systemic turnover changes, rather than a definitive characterization of global skeletal formation rates across all skeletal sites.

## 5. Conclusions

In conclusion, our preclinical model demonstrates that juvenile doxorubicin exposure inflicts substantial and enduring damage to the skeletal system, primarily characterized by sustained trabecular network failure and severe growth plate degeneration. This lasting skeletal fragility is not merely a consequence of acute cytotoxicity but rather stems from a pathologically uncoupled remodeling environment that firmly establishes itself during the recovery phase. Specifically, the transition from an acute mismatch in bone turnover to a sustained state of localized, osteoclast-driven bone resorption—coupled with a parallel decline in circulating formation markers—suggests a significant disruption in the bone remodeling balance. This environment appears to hinder the structural repair of the developing skeleton during the critical recovery window, potentially contributing to long-term skeletal fragility.

## Data Availability

The data presented in this study are available on request from the corresponding authors.

## Abbreviations

The following abbreviations are used in this manuscript:

ALL	Acute lymphoblastic leukemia
AML	Acute myeloid leukemia
BGP	Bone Gla protein
BMD	Bone mineral density
BTM	Bone turnover marker
BV/TV	Bone volume/Tissue volume
Conn.Dn	Connectivity density
DOX	Doxorubicin
EDTA	Ethylenediaminetetraacetic acid
ELISA	Enzyme-linked immunosorbent assay
Eu.N	Euler number
H&E	Hematoxylin and Eosin
i.v.	Intravenous
OC	Osteocalcin
PFA	Paraformaldehyde
PINP	Procollagen type I N-terminal propeptide
ROI	Region of interest
ROS	Reactive oxygen species
SMI	Structure model index
Tb.N	Trabecular bone number
Tb.Sp	Trabecular bone separation
Tb.Th	Trabecular bone thickness
TGF- β	Transforming growth factor beta
TRACP-5b	Tartrate-resistant acid phosphatase 5b
μCT	Micro-computed tomography
